# Geography Plays a More Important Role than Soil Composition on Structuring Genetic Variation of Pseudometallophyte *Commelina communis*

**DOI:** 10.3389/fpls.2016.01085

**Published:** 2016-07-22

**Authors:** Jiaokun Li, Hui Xu, Yunpeng Song, Lulu Tang, Yanbing Gong, Runlan Yu, Li Shen, Xueling Wu, Yuandong Liu, Weimin Zeng

**Affiliations:** ^1^School of Metallurgy and Environment, Central South University, ChangshaChina; ^2^School of Minerals Processing and Bioengineering, Central South University, ChangshaChina; ^3^College of Life Sciences, Central China Normal University, WuhanChina; ^4^School of Life Sciences, Central South University, ChangshaChina; ^5^State Key Laboratory of Hybrid Rice, College of Life Sciences, Wuhan University, WuhanChina

**Keywords:** *Commelina communis*, genetic structure, heavy metal pollution, local adaptation, microsatellites, pseudometallophytes

## Abstract

Pseudometallophytes are excellent models to study microevolution and local adaptation to soil pollution, as they can grow both on metalliferous and contrasting non-metalliferous soils. Although, there has been accumulating evidence for the effects of edaphic conditions and geographical isolation on the genetic structure of pesudometallophytes, it is still a difficult problem in evolutionary biology to assess their relative importance. In this study, we investigated the spatial patterns of genetic variability, population differentiation and genetic groups in pseudometallophyte *Commelina communis* with 12 microsatellite loci. Eight metallicolous and six non-metallicolous populations of *C*. *communis* were sampled from cupriferous sites and surrounding non-contaminated areas in China. Neither significant reduction in genetic diversity nor apparent founder and bottleneck effects were observed in metallicolous populations of *C. communis.* Based on Bayesian and Neighbor-Joining clustering analyses and a principal coordinates analysis, all sampled populations were found to be mainly separated into three genetic groups, corresponding well to their geographical locations rather than edaphic origins. Moreover, a significant and strong correlation between population genetic divergence and geographical distance were detected by Mantel test (*r* = 0.33; *P* < 0.05) and multiple matrix regression with randomization (MMRR; β_D_ = 0.57, *P* < 0.01). However, the effect of copper concentration on genetic patterns of *C*. *communis* was not significant (MMRR; β_E_ = -0.17, *P* = 0.12). Our study clearly demonstrated that the extreme edaphic conditions in metalliferous areas had limited effects on the genetic variability in *C*. *communis*. Geographic distance played a more important role in affecting the genetic structure of *C*. *communis* than soil composition did. In *C*. *communis*, the geographically disjunctive populations on metalliferous soils had multiple origins and evolved independently from nearby non-metallicolous populations.

## Introduction

Environmental stress experienced by living organisms plays an important role in local adaptation and evolution of new species (Hoffman and Parsons, 1991; Parsons, 2005; Nevo, 2011). Soils contaminated with high concentrations of heavy metals (metalliferous soils) are toxic and restrictive habitats for plants due to phytotoxicity (Antonovics et al., 1971; Wu, 1990; Ernst, 1996). The harsh edaphic conditions in metalliferous areas therefore have been considered as the major environmental determinants of plant survival and distribution (Cain, 1944; Rajakaruna, 2004). However, some plant species (known as metallophytes) that have evolved heavy metal-tolerance can colonize, survive, and reproduce in such conditions without suffering toxicity (Antonovics et al., 1971; Simon, 1978; Macnair, 1987; Baker et al., 2010). Pseudometallophytes, i.e., species growing on both metalliferous and non-metalliferous soils have been found to be the dominant plants in heavy metal contaminated areas (Ernst, 2006). Exploring the genetic variation and differentiation between metallicolous (M) and non-metallicolous (NM) populations in pseudometallophytes provides us an ideal opportunity to understand the processes of plant local adaptation and microevolution in the context of environmental pollution (Linhart and Grant, 1996; Vekemans and Lefèbvre, 1997; Mengoni et al., 2000, 2001; Meyer et al., 2010).

Genetic variation at intra- and inter-population level have been considered as the preconditions of plant local adaptation or evolution (Bone and Farres, 2001; Jiménez-Ambriz et al., 2006; Słomka et al., 2011). The extremely edaphic conditions on metal-contaminated areas can exert strong selection pressure and significantly influence the levels of genetic diversity within and among populations of pseudometallophytes (Macnair et al., 2000; Assunção et al., 2003; Dubois et al., 2003). In initial M populations, the number of metal tolerant individuals is assumed to be small (Macnair, 1987; Al-Hiyaly et al., 1993) and recently established populations on the metalliferous soils therefore may experience strong founder and bottleneck effects (Lefèbvre and Vernet, 1990; Linhart and Grant, 1996; Vekemans and Lefèbvre, 1997; Pollard et al., 2002). In multiple previous studies, significant reduction in genetic variability has been detected in populations of pseudometallophytes from metal contaminated sites (Nordal et al., 1999; Mengoni et al., 2001; Deng et al., 2007; Meyer et al., 2010; Babst-Kostecka et al., 2014). However, opposite results were also observed in some other pseudometallophytes, where the levels of genetic diversity were similar in both NM and M populations (Mengoni et al., 2000, 2006; Pauwels et al., 2005; Baumbach and Hellwig, 2007; Bizoux et al., 2008; Wójcik et al., 2013) or even higher in populations established in metal-contaminated soils (Słomka et al., 2011; Dresler et al., 2015). Such contrasting patterns suggest that soil composition is an important factor affecting the patterns of genetic variation in pesudometallophytes, but not the only one. To better understand the effects of edaphic conditions on the genetic variation in pesudometallophytes, other additional factors that can possibly influence the initial founder effects should be investigated, such as the level of selection pressure, the level of gene flow among populations, the successive mutation events, the geographic distribution patterns, and the plant reproductive systems (Ye et al., 2012; Babst-Kostecka et al., 2014). So far, it is still unclear how these factors, either individually or jointly, come into play during the colonization of pseudometallophytes.

Generally, geography plays an important role in population genetic divergence and speciation. The greater physical distance among populations will lead to the stronger internal genetic differentiation (Rieseberg and Willis, 2007). Heavy metal contaminated sites are featured for their patchy distributions and usually surrounded by non-contaminated mainland (Lefèbvre and Vernet, 1990). In such a case, populations from metalliferous soils should have closer genetic relationships with geographically adjacent NM populations than other distant conspecific M populations. Therefore, the hypothesis of multiple and independent origins for M populations of pesudometallophytes has been proposed (Schat et al., 1996; Mengoni et al., 2001; Pauwels et al., 2005; Quintela-Sabarís et al., 2010). However, extremely edaphic conditions can also cause the development of population differentiation (Bone and Farres, 2001). Under heavy metal stress, a small geographical interval between M and NM populations can result in significant genetic divergence or even sympatric speciation (Linhart and Grant, 1996; Rajakaruna, 2004). Although, both edaphic conditions and geography should contribute to shaping the spatial population structure of pesudometallophytes, it is still difficult to assess their relative importance on this event.

*Commelina communis* L. (Commelinaceae), commonly known as dayflower, is distributed extensively throughout China except for a few provinces, such as Qinghai, Hainan, Xinjiang, and Tibet (Hong and DeFillipps, 2000; Huang, 2001). This species can grow on both cupriferous habitats and surrounding non-metalliferous areas and has been considered as a typical pseudometallophyte (Tang et al., 1997, 1999, 2001; Ye et al., 2012). In the previous study conducted by Ye et al. (2012), the populations of *C*. *communis* from both metalliferous and nearby non-metalliferous soils mainly clustered into two groups, corresponding well to edaphic types rather than geographic locations (Ye et al., 2012). Cu contamination instead of geographic distance therefore had been considered as the major factor influencing the genetic structure of *C*. *communis*. However, it should be noted that the sampling sites for M populations in the previous research were relatively close and mainly concentrated in eastern China (Ye et al., 2012), the potential effect of geography might be underestimated.

To accurately evaluate the effects of edaphic conditions and geographical factors on the microevolutionary processes of pesudometallophytes, multiple replicates of M and NM populations separated by large geographic distances should be analyzed. In this study, 14 *C*. *communis* populations were sampled from three main regions in China, covering a much broader ranges of geographic distribution than the previous study (Ye et al., 2012). Twelve highly polymorphic microsatellite loci were used to estimate the genetic diversity and population structure of *C*. *communis* (Jarne and Lagoda, 1996; Sunnucks, 2000; Zhang and Hewitt, 2003; Selkoe and Toonen, 2006). The specific aims of this study were to: (1) assess the genetic variability and differentiation among populations of *C*. *communis* established in metalliferous and non-metalliferous soils; (2) test the relative importance of geographic distance and soil composition on shaping genetic differentiation of *C*. *communis* populations; (3) explore whether *C*. *communis* populations established in metalliferous and non-metalliferous soils had a single or multiple independent origins.

## Materials and Methods

### Plant and Soil Sampling

**FIGURE 1:**
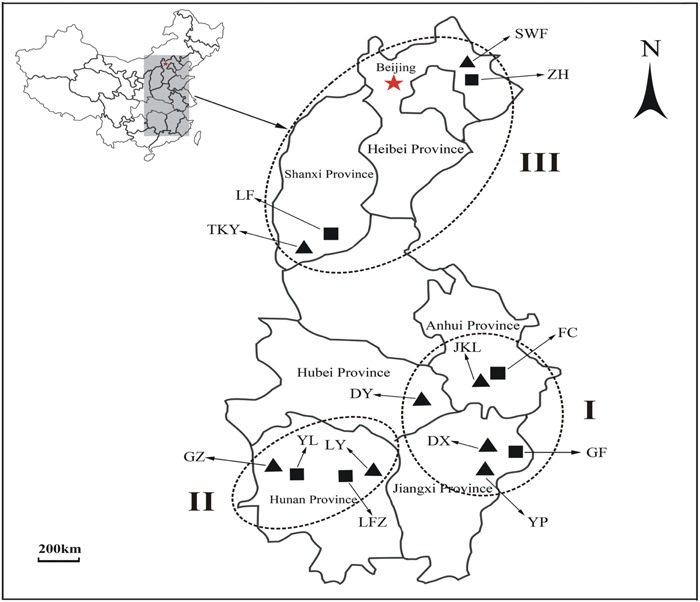
**The geographical locations of 14 *Commelina communis* populations in this study. ▲** Metalliferous populations of *C*. *communis*; ■ non-metalliferous populations of *C*. *communis.* I, II, and III represent three different sampling groups.

Eight natural copper mines located in different geographic areas of China were selected as sampling sites for M populations of *C*. *communis* (**Table 1** and **Figure 1**). Sampled M populations were discontinuously distributed in three geographical regions (I: east-central china, II: central China, and III: north China; **Figure 1**). Six NM populations were also sampled from nearby non-metalliferous soils. In each population, leaves of *C*. *communis* were collected from twenty individuals separated by at least 5 m and immediately dried in silica gel for genomic DNA extraction. In addition, 10 rhizospheric soil samples were randomly collected from 5 to 15 cm in depth at each sampling site.

**Table 1:** Locations of 14 populations of *Commelina communis*, and characterizations of soils associated with plants.

Edaphic type	Populations locality	Code	Latitude (N)	Longitude (E)	*n*	Concentration of Cu in soils (mg kg^-1^, mean ± SD)	
						Total Cu	Extractable Cu
M	Shouwangfen, Heibei	SWF	40°59′20′′	117°86′48′′	20	1796 ± 155	264 ± 35
M	Tongkuangyu, Shanxi	TKY	35°33′23′′	111°68′18′′	20	1393 ± 183	193 ± 42
M	Jinkouling, Anhui	JKL	30°90′75′′	117°82′11′′	20	1630 ± 201	220 ± 53
M	Daye, Hubei	DY	30°08′19′′	114°94′36′′	20	1480 ± 120	203 ± 28
M	Dexing, Jiangxi	DX	29°02′17′′	117°72′34′′	20	2650 ± 79	489 ± 15
M	Guzhang, Hunan	GZ	28°68′30′′	110°10′18′′	20	3400 ± 320	535 ± 89
M	Liuyang, Hunan	LY	28°21′74′′	113°56′29′′	20	1880 ± 420	339 ± 102
M	Yongping, Jiangxi	YP	28°21′49′′	117°77′31′′	20	2100 178	398 ± 40
	Mean ± SD					2041.1 ± 677.3^a^	330.1 ± 132.8^c^
NM	Zunhua, Hebei	ZH	40°22′27′′	117°99′64′′	20	50 ± 1.5	7.9 ± 0.2
NM	Linfen, Shanxi	LF	35°95′21′′	111°76′33′′	20	30 ± 3.8	4.8 ± 0.3
NM	Fanchang, Anhui	FC	31°08′50′′	118°22′35′′	20	25 ± 0.7	6.1 ± 0.1
NM	Guangfeng, Jiangxi	GF	28°46′58′′	118°19′13′′	20	80 ± 4.2	10.1 ± 0.2
NM	Yuanling, Hunan	YL	28°44′89′′	110°38′87′′	20	10 ± 2.3	2.1 ± 0.1
NM	Leifengzhen, Hunan	LFZ	28°19′52′′	112°84′10′′	20	15 ± 0.6	3.4 ± 0.1
	Mean ± SD					35 ± 26.1^b^	5.7 ± 2.9^d^

*M, metallicolous; NM, non-metallicolous; n, number of individuals per population. Data followed by the different letters in the same column indicate significant difference (*P* < 0.05).*

### Soil Chemical Analyses

Sampled soils were dried and sieved through a 2-mm mesh. The concentrations of total and extractable Cu in soils were analyzed using atomic absorption spectrometry (Hitachi, Japan). For the total Cu concentrations, prepared soils (three replicates per site) were cold-digested with HCl and HNO_3_ (3:1) for at least 12 h, then digested at 120°C for 2 h. For the extractable Cu concentrations, soil samples (three replicates per site) were extracted with diethylenetetraminepenta acid (DTPA) solution.

### DNA Extraction and Microsatellite Analysis

The genomic DNA was extracted from dried leaves of *C. communis* using the DNA extraction Kit (Qiagen, Hilden, Germany). The quality of extracted DNA was measured by NanoDrop spectrophotometer (Thermo Scientific, USA).

Polymorphism was assayed on each DNA sample with 12 microsatellite markers developed in our previous study (Supplementary Table S1; Li et al., 2015b). The amplification reaction was carried out in 20 μL reaction mixture, containing 30–50 ng genomic DNA, 7.5 μL of 2×*Ta*q PCR MasterMix (Tiangen, Beijing, China), 0.6 μM of each primer. Three fluorescent compounds (HEX, ROX or 6-FAM, Invitrogen, Carlsbad, CA, USA) were used to label the forward primers for the automated sequencers analysis (Supplementary Table S1). Amplification were carried out with an Eppendorf Mastercycler pro vapo protect thermocycler (AG, Hamburg, Germany), using the following thermocycling condition: initial denaturation at 95°C for 3 min, followed by 30 cycles of 30 s denaturation at 94°C, 30 s annealing at the optimal temperature (depending on each locus) and 1 min extension at 72°C, then 7 min at 72°C for the final extension. The ABI PRISM 3730XL DNA Sequencer (Applied Biosystems) and GeneMapper version 4.0 (Applied Biosystems) were used to separate the amplified products and determine the allele sizes, respectively.

### Data Analysis

#### Genetic Diversity

The errors and null alleles in the microsatellite data were tested by MICROCHECKER 2.2.3 (Van Oosterhout et al., 2004). The linkage disequilibrium between all pairs of locus was checked by GENEPOP 4.0 (Raymond and Rousset, 1995). The *P*-values were corrected with sequential Bonferroni correction (Rice, 1989). To test for signs of positive and balancing selection on these loci, the *F*_ST_-outlier approach (Beaumont and Nichols, 1996; Beaumont, 2005) implemented in LOSITAN (Antao et al., 2008) was used. The tests for outlier loci were performed using the stepwise mutation model with 100, 000 simulations and a confidence interval of 0.95. Loci that presented *F*_ST_ higher than the 95% confidence interval were considered candidates for divergent selection, and that presented *F*_ST_ lower than the 95% confidence interval were considered candidates for balancing selection. For each microsatellite locus, the observed (*H*_o_) and expected (*H*_e_) heterozygosity, the number of alleles (*N*_a_) and the Polymorphism Information Content (*PIC*) were calculated with PowerMarker v3.23 software (Liu, 2002). Meanwhile, the average number of alleles *N*_al_, *H*_o_, *H*_e_ in each population across all microsatellite loci were estimated with the same software. The allelic richness (*A*_r_) and the number of ‘private’ alleles (*N*_pr_) for each population was estimated by FSTAT 2.9.3.2 (Goudet, 1995) and MICROSAT (Minch et al., 1997), respectively.

The average levels of Cu concentration and genetic variation between M and NM populations were compared with independent- *t*-test in the SPSS statistical package (IBM, Corp., Armonk, NY, USA). To determine whether there was a significant association between the geographic location and genetic diversity in *C*. *communis* populations, multiple regression analyses were performed in the SPSS with latitude and longitude as covariates and M-NM as fixed factor. Complementarily, we performed paired *t*-test to compare levels of genetic diversity between nearby pairs of populations established in metalliferous and non-metalliferous soils (SWF-ZH, TKY-LF, JKL-FC, DX-GF, GZ-YL, and LY-LFZ).

#### Population Genetic Structure

To identify the patterns of genetic structure in *C*. *communis*, a Bayesian model-based clustering, regardless of an individual’s geographic origin, was performed in STRUCTURE to infer the number of potential genetic clusters (*K*) (Pritchard et al., 2000). The clustering was conducted with the admixture model and correlated allele frequencies using the 100, 000 burn-in time periods and 1, 000, 000 Markov Chain Monte Carlo (MCMC) iterations. The different number of clusters (K) from 1 to 14 was tested with 10 independent runs. The most likely number of *K* was estimated from the posterior probability of the log-likelihood distribution Pr (*X*|*K*) (Pritchard et al., 2000) and its second-order rate of change (Δ*K*) (Evanno et al., 2005).

**FIGURE 2:**
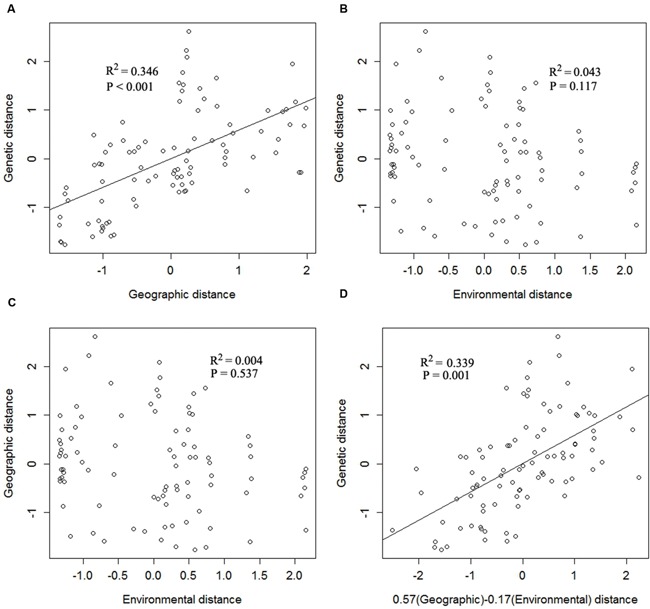
**Multiple matrix regression with randomization analysis (MMRR) scatter plots showing the relationships of **(A)** geographical and genetic distance, **(B)** environmental and genetic distance, **(C)** environmental and geographical distance, and **(D)** the inferred multiple regression for the effects of geographical (β_D_ = 0.57) and environmental distances (β_E_ = -0.17) on genetic distance**.

To assess the hierarchical partitioning of genetic variation in *C*. *communis*, an analysis of molecular variance (AMOVA) was performed with Arlequin 3.5.1 (Excoffier and Lischer, 2010). Two alternative population groups were examined: (1) a arrangement based on geographical locations in east-central China (Group I: JKL, DY, DX, YP, FC, GF) vs. sites in central China (Group II: GZ, LY, YL, LFZ) vs. sites in north China (Group III: SWF, TKY, ZH, LF; **Figure 1**); (2) a arrangement based on edaphic types with Cu-contaminated sites (Group M: SWF, TKY, JKL, DY, DX, GZ, LY, YP) vs. non-contaminated sites (Group NM: ZH, LF, FC, GF, YL, LFZ; **Figure 1**). In each analysis, 1, 000 permutations were used to test the significance levels of variance components. To test the correlation between the genetic (Pairwise *F*_ST_) and geographic distances among populations of *C*. *communis*, the Mantel test was conducted in Arlequin 3.5.1 with 10, 000 permutations (Mantel, 1967; Excoffier and Lischer, 2010). Pairwise *F*_ST_ values for each population comparison were calculated with FSTAT 2.9.3.2 (Goudet, 1995). The geographic distances between populations were measured as the straight-line distances according to their coordinates. Moreover, multiple matrix regression with randomization (MMRR) was used to explicitly test the role of environment (soil composition) vs. geography on shaping observed patterns of genetic differentiation. MMRR was implemented with 10, 000 permutations in R with the MMRR function script (Wang, 2013). The regression analysis was based on distance matrices of genetic and geographical and a matrix of dissimilarity in copper concentration between the studied populations.

#### Phylogeographic Relationships among Populations

To explore the genetic relationships among *C*. *communis* populations, a Neighbor-joining (NJ) tree was constructed with Cavalli-Sforza’s and Edwards chord distance (Dc) based on microsatellite allele frequencies (Takezaki and Nei, 1996; Goldstein and Pollock, 1997). Bootstrap (10, 000 replicates) was performed to evaluate the node robustness. In addition, a principal coordinates analysis (PCoA) based on pairwise genetic difference was implemented with GenALEx version 6.5 (Peakall and Smouse, 2012) to validate the naturally occurring genetic clusters.

#### Bottleneck Analysis

When a population undergoes a recent bottleneck, its reduced effective population size exhibits a correlated reduction in allele numbers and heterozygosity at polymorphic loci. However, the number of alleles in bottlenecked population is more severely affected than heterozygosity, i.e., the allelic diversity is reduced faster than the heterozygosity. Therefore, the bottlenecked population is predicted to show an excess of heterozygosity. To detect the evidence for apparent bottleneck events in populations of *C*. *communis*, the program BOTTLENECK v 1.2.02 was used (Cornuet and Luikart, 1996; Piry et al., 1999). The two-phase mutation model (TPM) that has been considered as the best model for microsatellite data in BOTTLENECK was adopted in our study (Di Rienzo et al., 1994; Piry et al., 1999). The proportion of single step mutation events was set to 95%, with a variance of 12 (Piry et al., 1999). Wilcoxon sign-rank test was used to test the significant heterozygosity excess (Cornuet and Luikart, 1996; Piry et al., 1999).

## Results

### Characterization of Soil Conditions

An overview of geographical locations and soil characteristics for the 14 *C*. *communis* populations were presented in **Table 1** and **Figure 1**. The average concentrations of total and extractable Cu in the metalliferous soils were significantly higher than those in non-metalliferous soils (**Table 1**). Compared to non- metalliferous areas, the average Cu concentrations was almost 50 times higher at the metalliferous sites (**Table 1**). In addition, large variances in Cu concentrations among the metalliferous sites were also detected. The highest values were reached up to 3400 ± 320 mg kg^-1^ for the total Cu and 535 ± 89 for the extractable Cu mg kg^-1^ at population GZ, whilst the lowest concentrations of total and extractable Cu were 1393 ± 183 and 193 ± 42 mg kg^-1^, respectively at population TKY.

### Polymorphism of Microsatellite Loci

Over the 12 SSR loci, no evidence of null alleles or scoring error was detected using MICROCHECKER. Deviations from linkage equilibrium were not significant after Bonferroni corrections (*P* > 0.0001). A total of 135 different alleles were found across all the 14 populations of *C*. *communis*. The number of alleles among polymorphic loci ranged from four to 28, and the average value per locus was 11.25 (Supplementary Table S1). For each locus, the observed (*H*_o_) and expected (*H*_e_) heterozygosity ranged from 0.552 to 0.905 and 0.567 to 0.913, with a mean value of 0.764 and 0.771, respectively (Supplementary Table S1). The *PIC* values of the microsatellite loci varied from 0.535 to 0.901 in our study (Supplementary Table S1). LOSITAN analyses revealed that no loci were under directional or balancing selection (**Supplementary Figure S1**).

### Population Genetic Diversity

*Commelina communis* showed high levels of genetic diversity (**Table 2**). The mean number of alleles (*N*_al_) for M and NM populations of *C*. *communis* ranged from 1.833 (TKY) to 6.500 (DY) and 2.500 (LFZ) to 5.167 (GF), respectively. No significant associations between genetic diversity and population latitude (*P* = 1.0) and longitude (*P* = 0.9) were detected by multiple regression analyses. The parameters of average gene diversity indices were relatively high in most populations from metalliferous soils, with the average values of 3.558 for *A*_r_, 0.440 for *H*_o_ and 0.555 for *H*_e_ (**Table 2**). There were no significant differences between M and NM populations in average *A*_r_ (*t* = 0.03, *P* = 0.976), *H_o_* (*t* = 1.04, *P* = 0.319), and *H_e_* (*t* = -0.23, *P* = 0.823; **Table 2**). Paired *t*-test considering nearby populations established in metalliferous and non-metalliferous soils did not show either significant difference in *A*_r_ (*t* = -1.12, *P* = 0.312), *H_o_* (*t* = 1.23, *P* = 0.272), or *H_e_* (*t* = -1.33, *P* = 0.239). Private alleles were detected in both M and NM populations of *C*. *communis* and no significant differences were observed in average *N*_pr_ between M and NM populations (*t* = 1.84, *P* = 0.091; **Table 2**). However, the total number of private alleles within M populations (24) was almost fourfold that within NM populations (7). The populations with most private alleles were DY (7) and JKL (5) (**Table 2**).

**Table 2:** Genetic diversity within the 14 populations of *C*. *communis.*

Edaphic type	Populations	*A*_r_	*N*_al_	*H*_o_	*H*_e_	*N*_pr_
M	SWF	1.950	2.000	0.317	0.362	0
M	TKY	1.808	1.833	0.350	0.218	2
M	JKL	4.611	4.667	0.633	0.702	5
M	DY	6.403	6.500	0.539	0.826	7
M	DX	2.397	3.833	0.517	0.624	2
M	GZ	4.287	4.500	0.421	0.645	3
M	LY	2.103	2.500	0.406	0.415	3
M	YP	4.904	5.167	0.333	0.651	2
	Mean ± SD	3.558 ± 1.718^a^	3.875 ± 1.654^b^	0.440 ± 0.113^c^	0.555 ± 0.203^d^	3.0 ± 2.138^e^
NM	ZH	3.098	3.167	0.333	0.498	0
NM	LF	3.600	3.667	0.423	0.544	0
NM	FC	4.561	4.667	0.517	0.663	3
NM	GF	4.877	5.167	0.255	0.735	2
NM	YL	2.633	2.833	0.394	0.542	0
NM	LFZ	2.432	2.500	0.367	0.475	2
	Mean ± SD	3.533 ± 1.008^a^	3.667 ± 1.054^b^	0.382 ± 0.088^c^	0.576 ± 0.101^d^	1.167 ± 1.329^e^

*N_*al*_, the average number of alleles; *A*_*r*_, allelic richness; *H*_*o*_, observed heterozygosity; *H*_*e*_, expected heterozygosity; *N*_*pr*_, private alleles per population; Data followed by the same letters in the same column indicate no significant difference (*P* > 0.05).*

### Population Genetic Structure

The overall pairwise *F*_ST_ value was 0.325 (95% confidence interval: 0.213–0.443). Except three pairs of populations (FC and JKL, GF and YP, LFZ and LY), all the values of pairwise *F*_ST_ were significantly different from 0 (*P* < 0.05; Supplementary Table S2). The Mantel test indicated that there was a positive correlation between the genetic and geographical distance (*r* = 0.33; *P* < 0.05), indicating that geography is an important factors affecting the genetic structure of *C*. *communis*. Moreover, the results of MMRR revealed that the genetic distance had a significant correlation with geographic distance (β_D_ = 0.57, *P* < 0.01). However, the effect of environmental factor (copper concentration) on genetic patterns of *C*. *communis* was not significant (β_E_ = -0.17, *P* = 0.12; **Figure 2**).

**FIGURE 3:**
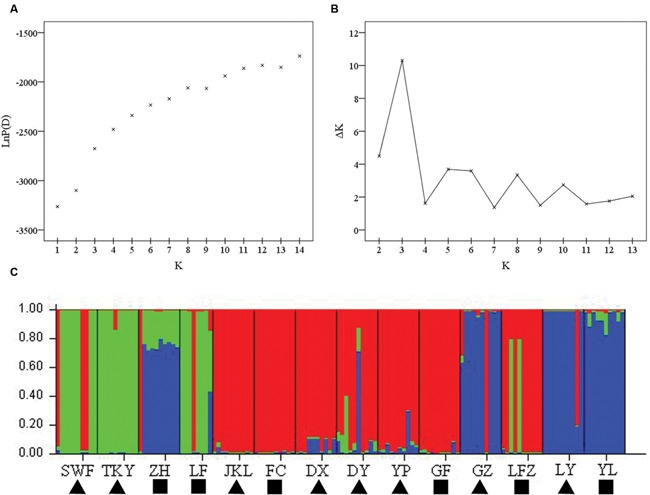
**The Bayesian analysis results of *C*. *communis* based on SSR data. (A)** Plot of mean posterior probability LnP(D) values of each *K*; **(B)** STRUCTRE estimation of the number of populations for K ranging 1–14 by delta *K*-value (Δ*K*); **(C)** the result of the assignment of individuals for *K* = 3. Individuals are represented as thin vertical lines partitioned into segments corresponding to the inferred genetic cluster as indicated by color and populations were marked on the bottom. The abbreviations of population name are same as **Table 1**.

**FIGURE 4:**
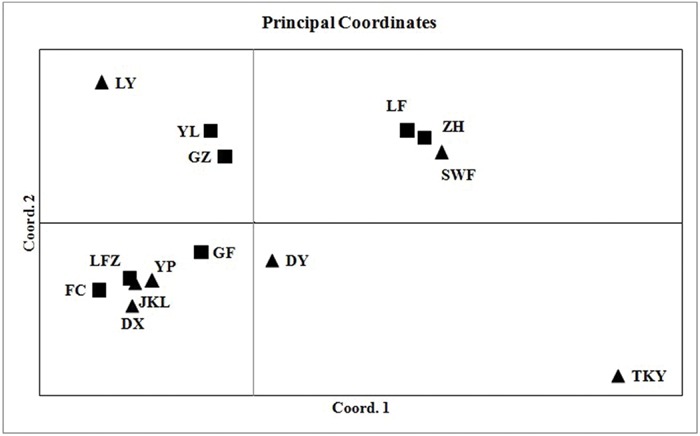
**Principal coordinates analysis illustrating genetic differences among 14 populations of *C. communis*.** Coordinate 1 is accounted for 42.57% and coordinate 2 for 28.77% of the total variation among populations. The abbreviations of population name are same as **Table 1**.

**FIGURE 5:**
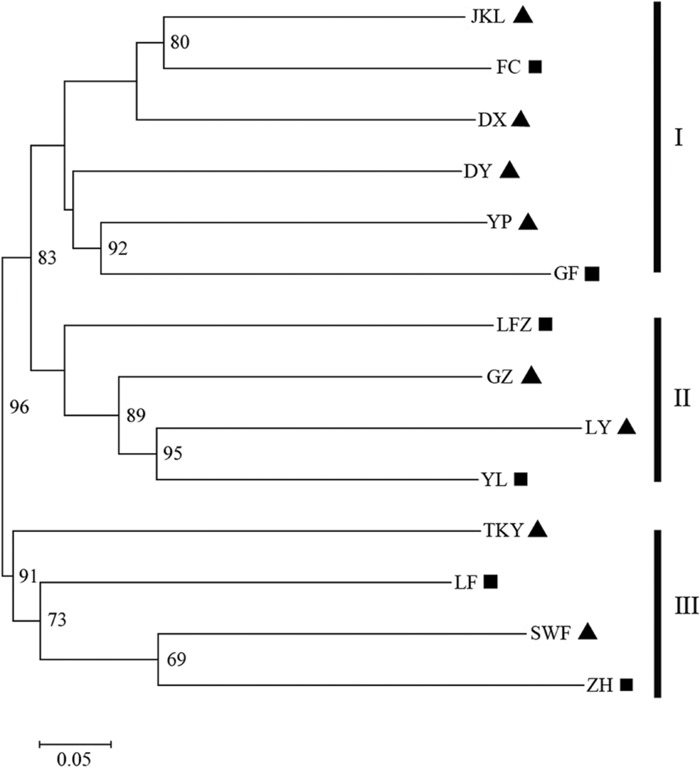
**The Neighbor-joining (NJ) tree of *C*. *communis* accessions based on Cavalli-Sforza’s chord distance.** Number at nodes represent the frequency (expressed as a percentage) at which a node occurred among 10, 000 replications. Bootstrap values higher than 60% from 1000 replicates are shown above the branches. The abbreviations of population name are same as **Table 1**.

The number of subpopulations (K) was identified based on maximum likelihood and Δ*K* values. A clear peak in the value of Δ*K* was detected at *K* = 3 (**Figures 3A,B**). In general, three gene pools were evident. The north China populations were predominantly composed of a single gene pool, whereas the other two gene pools were mostly detected in the east-central China populations and the central China populations (**Figure 3C**). In addition, a general picture of admixture genotypes also emerged in our analysis and the ancestry of several genotypes in the 14 populations could be traced to more than one gene pool (**Figure 3C**). Several genotypes form one population shared alleles with other populations, indicating that these populations likely maintained frequent gene flow (**Figure 3C**). The PCoA results supported the findings from STRUCUTRE, demonstrating that genetic clustering of populations was in concordance with their geographic origins (**Figure 4**). However, there was an overlap of population LFZ from central China with population JKL from east-central China, indicating that intensive gene flow might occur between these two populations (**Figure 4**). The relationships between populations in the NJ tree showed that populations sampled from the metalliferous and non-metalliferous sites were not clearly separated, but divided into three distinct clusters, broadly corresponding to three geographical areas (**Figure 5**). All the populations located in east-central China formed one cluster (cluster I). The populations from central China and north China comprised another two major clusters (cluster II and cluster III). *C*. *communis* populations with close geographic distances had relatively low genetic divergence (**Figure 5**).

The results of AMOVA showed that most of the genetic variability occurred within populations of *C*. *communis* (61.51 and 64.16% of total variance for geographic and edaphic grouping, respectively), although variation among subpopulations were significant in both groupings (*P* < 0.001; **Table 3**). Grouping of the individual samples according to geographical distribution allowed 10.47% of the total variation (*P* < 0.001) to be accounted for by differences among the three geographic origins (**Table 3A**). Edaphic separations, however, showed little contribution to the total genetic variation (2.26%, *P* = 0.95; **Table 3B**).

**Table 3:** The results of analysis of molecular variance (AMOVA) for *C*. *communis* populations based on SSR data (under two alternative grouping of populations: geographical and strictly edaphic).

Source of variation	df	Sum of squares	Variance	% of total	*P*-value
**(A) Geographical partition (Group I vs. Group II vs. Group III)**					
Among groups	2	160.822	0.272	10.47	<0.001
Among populations within groups	11	337.598	0.727	28.02	<0.001
Within populations	546	871.925	1.597	61.51	<0.001
Total	559	1370.345	2.596		
**(B) Edaphic partition (Group M vs. Group NM)**					
Between groups Among populations within groups Within populations Total	1 12 546 559	24.086 474.333 871.925 1370.345	0.056 0.836 1.597 2.489	2.26 33.58 64.16	0.95 <0.001 <0.001

*Group I: JKL, DY, DX, YP, FC, GF; Group II: SWF, TKY, ZH, LF; Group III: GZ, LY, YL, LFZ; Group M: SWF, TKY, JKL, DY, DX, GZ, LY, YP; Group NM: ZH, LF, FC, GF, YL, LFZ.*

### Genetic Evidence for Population Bottlenecks

Bottleneck analysis under the TPM showed that there were no significant heterozygosity excesses in the 14 *C*. *communis* populations (*P* > 0.05, **Table 4**), indicating that all populations sampled from metalliferous areas did not experience apparent founder and bottleneck effects during their recent colonization.

**Table 4:** The results of bottleneck estimate in *C*. *communis* populations.

Edaphic type	Populations	*P*-value (Wilcoxon test)
M	SWF	0.781
M	TKY	0.813
M	JKL	0.219
M	DY	0.552
M	DX	0.219
M	GZ	0.922
M	LY	0.500
M	YP	0.578
NM	ZH	0.422
NM	LF	0.945
NM	FC	0.578
NM	GF	0.281
NM	YL	0.219
NM	LFZ	0.784

*Probabilities from tests for population bottlenecks in 14 populations of *C*. *communis* under the two-phase mutation model (TPM) in BOTTLENECK software.*

## Discussion

Our data showed that severe soil conditions at metalliferous areas did not significantly affect the levels of genetic variability in *C*. *communis*. The populations of *C*. *communis* from metalliferous soils had not undergone any apparent founder and bottleneck events during their colonization history. All sampled *C*. *communis* populations mainly clustered into three groups, corresponding well to their geographical locations rather than edaphic origins. Geographically distant M populations showed closer genetic similarity to their nearby NM populations than to each other. Both Mantel and MMRR analyses suggested that geographic distance played a more important role in determining the population structure of *C*. *communis* than soil composition did. Moreover, all Cu-contaminated populations branched into separated genetic groups, supporting the hypothesis of multiple and independent origins of M populations in *C*. *communis*.

### Microsatellite Variation in Populations of *C*. *communis*

Heavy metal tolerance is a fundamental condition for pseudometallophytes to survive on metalliferous soils. The evolutionary scenario assumes that metal-tolerant genotypes initially have low frequencies in natural populations (Al-Hiyaly et al., 1993); therefore, significant reduction in genetic diversity should be expected in the founder populations on metalliferous areas (Lefèbvre and Vernet, 1990; Wu, 1990). In many pseudometallophyte species, e.g., *Biscutella laevigata*, *Arabidopsis halleri*, *Silene paradoxa* and *Sedum alfredii*, the levels of genetic diversity in M populations were significantly reduced due to strong founder and bottleneck effects (Mengoni et al., 2001; Deng et al., 2007; Meyer et al., 2010; Ye et al., 2012; Babst-Kostecka et al., 2014). However, contrary to the aforementioned assumption, the levels of genetic diversity were not significantly reduced in M populations of *C*. *communis* (**Table 2**). For example, the DY population originating from Cu-contaminated sites had the highest number of private alleles as well as the highest value of genetic diversity indices (**Table 2**). Moreover, in contrast to other studies (Pauwels et al., 2006; Deng et al., 2007; Meyer et al., 2010; Ye et al., 2012), our large-scale investigation indicated that all populations sampled from Cu-contaminated sites did not experience apparent founder and bottleneck effects (**Table 4**). These results suggested that the selective pressure caused by Cu contamination might be not perceivably stronger for *C*. *communis* populations on metalliferous soils, or its effect on genetic variability had been balanced by other factors.

Gene flow has been considered as an important factor affecting the genetic variability of M populations in pseudometallophytes (Muller et al., 2004; Pauwels et al., 2005; Bizoux et al., 2008). The spatial genetic structure of *C*. *communis* could be significantly determined by the interplay and balance between local genetic drift and gene flow. The flowers of *C*. *communis* lack nectaries, and generalist pollinators, such as flies and bees, have been reported as their main pollinators (Faden, 1992, 2000; Ushimaru et al., 2007; Li et al., 2015a). With the aid of insect pollination, the levels of gene flow between M populations and adjacent NM populations of *C*. *communis* could be significantly increased and the initial founder and bottleneck effects therefore might be partly eroded by the successive gene flow. In the present study, shared ancestry in *C*. *communis* populations was detected by STRUCTURE (**Figure 3C**). Several populations shared alleles with other populations in spite of their far geographic distance. For example, individuals from population ZH located in north-China had inferred ancestry from east-central and central China. Similarly, mixed inferred ancestry was also found in population LFZ (**Figure 3C**). The population LFZ from central China had a close genetic relationship with east-central China populations (**Figure 4**). There was an overlap of population LFZ from non-metalliferous soils with metalliferous population JKL from east-central China (**Figure 4**), indicating that intensive gene flow might occur. Moreover, the AMOVA results showed that geography only accounted for 10% of the total genetic variation in our study (**Table 3**). Thus, all these results suggested that gene flow might play an important role in maintaining high genetic diversity in populations of *C*. *communis*. In addition, frequent nuclear DNA mutations in the M populations also could increase the population genetic diversity and reverse the initial founder and bottleneck effects (Mengoni et al., 2001; Dresler et al., 2015). Of all *C. communis* populations, the DY population sampled from Cu contaminated soils had the greatest number of private alleles. In addition, the M opulations with higher number of private alleles usually showed higher level of genetic variability in our study (**Table 2**), suggesting that genomic mutations may enhance the levels of genetic diversity in M populations of *C. communis*. However, we have to keep in mind that our study was based on only a small set of randomly chosen microsatellites. Because SSRs are neutral markers (**Supplementary Figure S1**) and may also be in disequilibrium with Cu selected gene, such mutations could be just replenishing genetic diversity in neutral regions of the genome and the necessary mutations for coping with Cu might be still at low frequency in M populations. Therefore, the overall genomic effect of the bottleneck might be small and quickly erased by gene flow from nearby NM populations or from other M populations. It also should be pointed out that *C*. *communis* is an out-crossing species, which can produce a lot of seeds every year. Each plant produces 5–80 bisexual flowers and each fruit has 2 elliptic, or 1 elliptic and 2 semi-elliptic, or 4 semi-elliptic seeds (Hong and DeFillipps, 2000). Because the mature seeds easily drop on the ground from the dehiscent fruits (Li et al., 2015a), the buried seeds in the soil therefore could be stored and form a seed bank. Seed banks have been suggested as the genetic reservoirs, which can maintain genotypes overtime and contain significantly larger amounts of genetic diversity than expected (Mandák et al., 2012). However, genetic consequences of a seed bank may vary among species and/or populations as suggested by Mahy et al. (1999), and more detailed studies are needed in the future to assess the importance of the seed bank in shaping genetic diversity and structure in *C*. *communis*.

### Genetic Differentiation between M and NM Populations of *C*. *communis*

Previous studies have shown that edaphic conditions (soil metal contamination) exert more influence than geographic distance on the genetic structure of pseudometallophytes (Deng et al., 2007; Parisod and Christin, 2008; Słomka et al., 2011; Ye et al., 2012; Babst-Kostecka et al., 2014). In contrast to previous studies, our investigation showed that the population structure of *C*. *communis* was mainly affected by geography rather than copper exposure. A significantly high level of genetic variability was found to be derived from the group of geographic partitions (*P* < 0.001), while that derived from the group of contaminated vs. non-contaminated populations was not significant (*P* = 0.95; **Table 3**). Both Mantel and MMRR analyses also indicated that the geographic distance rather than soil composition had a strong influence on genetic differentiation among populations of *C*. *communis* (**Figure 2**). In addition, M and NM populations were not clearly separated, but divided into three distinct clusters, broadly corresponding well with their geographical locations rather than edaphic types. Geographically adjacent *C*. *communis* populations showed relatively low genetic divergence, compared to populations far apart (**Figure 5**; Supplementary Table S2). Therefore, our data indicated that geography exerted more impact than copper exposure on the genetic structure of *C*. *communis.* Ye et al. (2012), however, suggested that Cu contamination played a more important role in shaping the population structure of *C*. *communis* than geographic distance. It should be noted that the average of total Cu concentrations (5404 ± 2151) and their extractable fractions (1196 ± 688) at metalliferous sites in Ye et al. (2012) are much higher than those in our study (**Table 1**), which may increase diverging selection and reduce gene flow between their study M and NM populations of *C*. *communis*. Moreover, ecogeographic distance has long been regarded as the most important factors to affect the gene flow in plants (Rieseberg and Willis, 2007). Although, we did find evidence of genetic differentiation between populations of *C*. *communis* likely due to the geographic distance, this is not an impervious barrier. Both STRUCTURE and PCoA results indicated a possible gene flow among populations in different regions (**Figures 3** and **4**). The population structure of *C*. *communis* was analyzed at a relatively small geographic scale in the previous research, concentrated and mainly limited in eastern China (Ye et al., 2012). The relatively close geographic distances among M populations of *C*. *communis* may mask the contribution of geography (Ye et al., 2012).

Because the metalliferous sites are often fragmented, the hypothesis of multiple and independent origins of M populations has been proposed and validated in some pseudometallophytes (Schat et al., 1996; Vekemans and Lefèbvre, 1997; Mengoni et al., 2001; Pauwels et al., 2005; Quintela-Sabarís et al., 2010). For *C*. *communis*, the geographically disjunctive M populations in our study clearly distributed into three distinct genetic clusters rather than one (**Figures 3–5**). All populations of *C*. *communis* from Cu-contaminated soils have closer genetic relationships with geographically adjacent NM populations than other distant conspecific M populations (**Figures 4** and **5**). Thus, our data indicated that the widely geographic distribution of M populations in *C*. *communis* today more likely evolved independently from nearby NM populations rather than from one single tolerant ancestral population (Ye et al., 2012).

## Conclusion

This study clearly demonstrated that the extreme edaphic conditions in metalliferous areas had limited effects on the genetic variability in *C*. *communis*. The populations of *C*. *communis* from metal contaminated sites have not experienced apparent historical founder and bottleneck events during their recent colonization. The high levels of genetic diversity in the M populations of *C*. *communis* may be resulted from extensive gene flow between neighboring NM populations or geographically close M populations, high frequency of genetic mutations, and/or a high number of tolerant plants in the initially established populations. The patterns of genetic structure in *C*. *communis* were primarily influenced by geographic distance rather than soil composition. Thus, we speculate that geography plays a more important role than edaphic condition in shaping the genetic structure of *C*. *communis*. In addition, the widely distributed M populations of *C*. *communis* may have evolved independently from multiple origins.

## Author Contributions

JL and WZ designed the study. HX and YS collected the data. JL, LT, YG, YL analyzed the data. JL and WZ drafted the manuscript. LS, RY, and XW critically revised the manuscript. All authors read and approved the final version of the manuscript.

## Conflict of Interest Statement

The authors declare that the research was conducted in the absence of any commercial or financial relationships that could be construed as a potential conflict of interest.

## Supplementary Material

FIGURE S1 **Assessment of outlier SSR loci using LOSTIAN software at the 95% threshold**. In red, the confidence area for candidate loci potentially under positive selection; in grey, the confidence area for neutral loci; in yellow, the confidence area for candidate loci potentially under balancing selection.
