# Supplementary figures and images for: Geography Plays a More Important Role than Soil Composition on Structuring Genetic Variation of Pseudometallophyte Commelina communis
Source: Front Plant Sci. 2016 Jul 22;7:1085. doi: 10.3389/fpls.2016.01085 (PMC4956667; doi:10.3389/fpls.2016.01085)

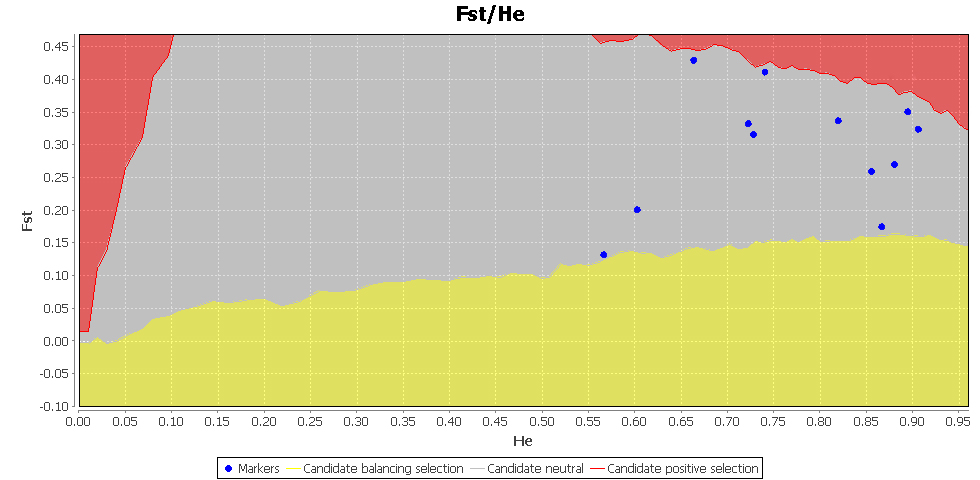

Supplement: FIGURE S1 — Assessment of outlier SSR loci using LOSTIAN software at the 95% threshold. In red, the confidence area for candidate loci potentially under positive selection; in grey, the confidence area for neutral loci; in yellow, the confidence area for candidate loci potentially under balancing selection. [file Image_1.JPEG]
