# Supplementary material for: Geography Plays a More Important Role than Soil Composition on Structuring Genetic Variation of Pseudometallophyte Commelina communis
Source: Front Plant Sci. 2016 Jul 22;7:1085. doi: 10.3389/fpls.2016.01085 (PMC4956667; doi:10.3389/fpls.2016.01085)
Supplement: Supplementary file 2 [file Table_1.DOCX]

**TABLE S1 Characteristics and genetic diversity of the 12 microsatellite loci used in 14 populations of *C*. *communis.***

| Locus | Primer sequence (5’-3’) | Repeat motif | Allele size range (bp) | *T*_a_ (°C) | Fluorescent dye | GenBank accession no. | *N*a | *H*_o_ | *H*_e_ | *PIC* |
| --- | --- | --- | --- | --- | --- | --- | --- | --- | --- | --- |
| YP5 | F: CACGGCTGATGGGAAGTTG  R: CTATGCATGCTTGGTTGAT | (CT)_7_ | 164-204 | 54 | 6-FAM | KJ647361 | 7 | 0.668 | 0.665 | 0.621 |
| YP6 | F: AAGCATTCTCCATTGTAAA  R: AGAAATGTGGGGGATAAA | (CT)_10_ | 102-138 | 55 | 6-FAM | KJ647362 | 9 | 0.717 | 0.720 | 0.706 |
| YP10 | F:AAGCACTCTCAACTGAAAAT  R:CAGTACAAGGAGACATAGA | (TC)_13_ | 133-157 | 55 | ROX | KJ647366 | 13 | 0.860 | 0.859 | 0.805 |
| YP14 | F: GCTTTTAGGATAACTTCAAC  R: GAGGACATTTAGGGCACAT | (TC)_10_ | 202-218 | 55 | HEX | KJ647370 | 9 | 0.738 | 0.743 | 0.727 |
| YP18 | F: CCTTTTCAACGCATATACA  R: TCTTCCAGGATCAAGTCAA | (CT)_13_ | 99-127 | 55 | HEX | KJ647374 | 13 | 0.862 | 0.867 | 0.819 |
| YP28 | F: TTAACGTTCACCTCACATG  R: GGCTTATATCAAATCCTACT | (CT)_15_ | 208-239 | 55 | 6-FAM | KJ647384 | 4 | 0.552 | 0.567 | 0.535 |
| YP31 | F: AAATTCAAATTCGATAGCA  R: AGAGTTTATATTTTGTGGA | (TC)_7_…(CT)_6_ | 84-120 | 56 | ROX | KJ647387 | 11 | 0.803 | 0.820 | 0.798 |
| YP33 | F: ATTATCTCTATGCCATCTCC  R: TTCTCTCCCTACACTTTTCT | (AG)_18_ | 295-319 | 55 | HEX | KJ647389 | 9 | 0.719 | 0.729 | 0.712 |
| YP34 | F: GCGTATTCAACCTGACAAAC  R: ACAAGAAGGGACATCAAAA | (TC)_16_ | 161-191 | 56 | ROX | KJ647390 | 14 | 0.887 | 0.899 | 0.853 |
| YP36 | F: CTCCCTTGTCCTCCCTCTTC  R: GGACTTTTTGGTTATTTGGT | (CT)_14_ | 194-206 | 57 | ROX | KJ647392 | 4 | 0.597 | 0.601 | 0.586 |
| YP37 | F: CAACAAATTGTTTTGAGAAT  R: AAGTGGTGTCTAACGATTGC | (CA)_7_…(AG)_16_ | 205-231 | 54 | ROX | KJ647393 | 14 | 0.858 | 0.872 | 0.834 |
| YP38 | F: ATATTTATTCCTTTTTCCCC  R: TATCATCACAAGCTCGCTAC | (TC)_14_ | 126-178 | 58 | 6-FAM | KJ647394 | 28 | 0.905 | 0.913 | 0.901 |
| Mean |  |  |  |  |  |  | 11.25 | 0.764 | 0.771 | 0.741 |
| Min. |  |  |  |  |  |  | 4 | 0.552 | 0.567 | 0.535 |
| Max. |  |  |  |  |  |  | 28 | 0.905 | 0.913 | 0.901 |
