# Supplementary material for: Geography Plays a More Important Role than Soil Composition on Structuring Genetic Variation of Pseudometallophyte Commelina communis
Source: Front Plant Sci. 2016 Jul 22;7:1085. doi: 10.3389/fpls.2016.01085 (PMC4956667; doi:10.3389/fpls.2016.01085)
Supplement: Supplementary file 3 [file Table_2.DOCX]

**TABLE S2 Pairwise *F*_ST_ values between all populations (lower-left matrix) of *C*. *communis* and their significance (upper-right matrix).**

| Populations | SWF | TKY | JKL | DY | DX | GZ | LY | YP | ZH | LF | FC | GF | YL | LFZ |
| --- | --- | --- | --- | --- | --- | --- | --- | --- | --- | --- | --- | --- | --- | --- |
| SWF | 0 | * | * | * | * | * | * | * | * | * | * | * | * | * |
| TKY | 0.628 | 0 | * | * | * | * | * | * | * | * | * | * | * | * |
| JKL | 0.379 | 0.475 | 0 | * | * | * | * | * | * | * | NS | * | * | * |
| DY | 0.305 | 0.324 | 0.082 | 0 | * | * | * | * | * | * | * | * | * | * |
| DX | 0.447 | 0.482 | 0.103 | 0.096 | 0 | * | * | * | * | * | * | * | * | * |
| GZ | 0.400 | 0.530 | 0.202 | 0.157 | 0.269 | 0 | * | * | * | * | * | * | * | * |
| LY | 0.588 | 0.686 | 0.336 | 0.281 | 0.355 | 0.343 | 0 | * | * | * | * | * | * | NS |
| YP | 0.443 | 0.545 | 0.091 | 0.066 | 0.174 | 0.200 | 0.412 | 0 | * | * | * | NS | * | * |
| ZH | 0.124 | 0.524 | 0.322 | 0.203 | 0.383 | 0.260 | 0.450 | 0.318 | 0 | * | * | * | * | * |
| LF | 0.244 | 0.195 | 0.295 | 0.220 | 0.355 | 0.274 | 0.514 | 0.304 | 0.333 | 0 | * | * | * | * |
| FC | 0.405 | 0.506 | 0.050 | 0.109 | 0.064 | 0.228 | 0.353 | 0.106 | 0.343 | 0.325 | 0 | * | * | * |
| GF | 0.359 | 0.460 | 0.113 | 0.070 | 0.213 | 0.204 | 0.321 | 0.048 | 0.283 | 0.284 | 0.172 | 0 | * | * |
| YL | 0.454 | 0.562 | 0.222 | 0.177 | 0.256 | 0.099 | 0.321 | 0.265 | 0.260 | 0.362 | 0.255 | 0.247 | 0 | * |
| LFZ | 0.473 | 0.608 | 0.233 | 0.231 | 0.322 | 0.286 | 0.041 | 0.238 | 0.405 | 0.447 | 0.249 | 0.274 | 0.373 | 0 |

* P < 0.05; NS, nonsignificant population differentiation. *F*_ST_, pairwise comparisons between populations of *C. communis* estimated from 12 microsatellite loci
